# Diffuse large B-cell lymphoma with clear cell morphology – a close mimic of clear cell carcinoma: A case report

**DOI:** 10.1097/MD.0000000000039919

**Published:** 2024-10-04

**Authors:** Guiyun Li, Qiang Feng, Jian Shen, Zhiyuan Wang, Yilong Huang, Huan Luo, Li Bian

**Affiliations:** a Department of Pathology, The First Affiliated Hospital of Kunmming Medical University, Kunming, Yunnan, China; b Department of Pathology, 920th Hospital of Joint Logistics Support Force, Kunming, China; c Department of Imaging, The First Affiliated Hospital of Kunmming Medical University, Kunming, Yunnan, China.

**Keywords:** case report, clear cell, diffuse large B-cell lymphoma, gene rearrangement, immunohistochemistry

## Abstract

**Rationale:**

In this case, the tumor cells were epithelioid, with translucent cytoplasm, well-defined cell borders, moderate size, rounded or ovoid nuclei, and small nucleoli visible. Based on this morphological feature, we first considered clear cell carcinoma, but the epithelial-related immunohistochemical results don’t support the diagnosis. Ultimately, based on lymphoma-associated immunohistochemical results and gene rearrangement assays, the final diagnosis of a clear cell variant of DLBCL. Therefore, clinicians and pathologists are reminded that DLBCL with clear cell morphology is rare.

**Patient concerns:**

A case of a 44-year-old patient who presented with a 1-year’s history of an oral tumor that had recently increased in size. Computed tomography (CT) showed an osteolytic lesion with soft tissue density in the right body of mandible with bone destruction in the cortical plates on both buccal and lingual sides.

Test results: Immunohistochemistry include vimentin, LCA, CD10, CD20, CD38, B-cell lymphoma (Bcl)-2, multiple myeloma oncogene (MUC)-1, CD79a were strongly expressed, while the tissue was negative for the rest of epithelium and other mesenchymal antibodies. Detection of Ig and TCR gene arrangements showing the presence of B-cell monoclonal rearrangement (DH7-JH) in the tumor.

**Diagnosis:**

The final diagnosis was germinal center B cell-like (GCB) diffuse large B lymphoma of clear cell type.

**Interventions:**

The patient was treated with chemotherapy.

**Outcomes:**

The patient was undergoing chemotherapy and has been followed up for over 1 year.

**Lessons:**

Diffuse large B-cell lymphoma with clear cell morphology is a rare tumor, and its diagnosis mainly relies on pathological findings, immunohistochemistry, gene rearrangement.

## 1. Introduction

Diffuse large B cell lymphoma (DLBCL) is the hematological malignancy, with an annual incidence exceeding 100,000 cases worldwide.^[1]^ DLBCL is characterized by significant heterogeneity at the clinical, genetic, molecular, immunophenotypic, morphological features, treatment responses and survival.^[2]^ Despite the new classification of DLBCL in the 5th edition of the WHO, there are still many cases that are biologically and clinically heterogeneous and are collectively referred to as DLBCL not otherwise specified (NOS). The clear cell diffuse large B-cell lymphoma as a special type and is still classified as Not Otherwise Specified, lymphomas with clear cell morphology occur in sites other than lymph nodes and are not easily considered for diagnosis. In this report, we present a case of primary DLBCL with clear cell morphology mimics clear cell carcinoma the cheek, and the diagnostic challenge of DLBCL with morphology that can lead to misdiagnosis if not properly assessed.

## 2. Case presentation

**Figure 1.**
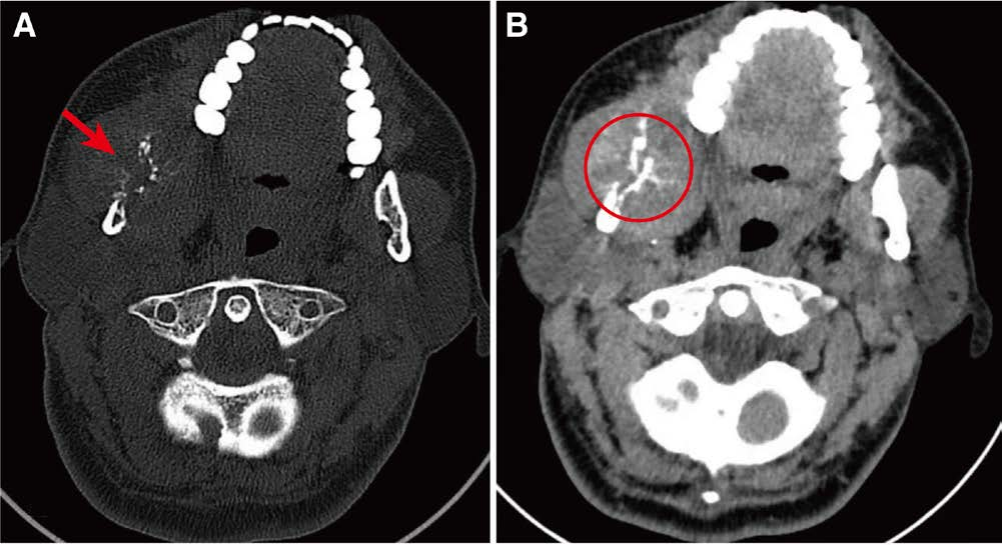
Computed tomography scans. (A) The bone window showed bone destruction of right mandible (red arrow). (B) The soft tissue window showed a soft tissue mass in the right mandible with radiolucent high density and distended growth with blurred margins, measuring approximately 4.8 cm × 3.8 cm × 4.9 cm at the largest level (red circle).

**Figure 2.**
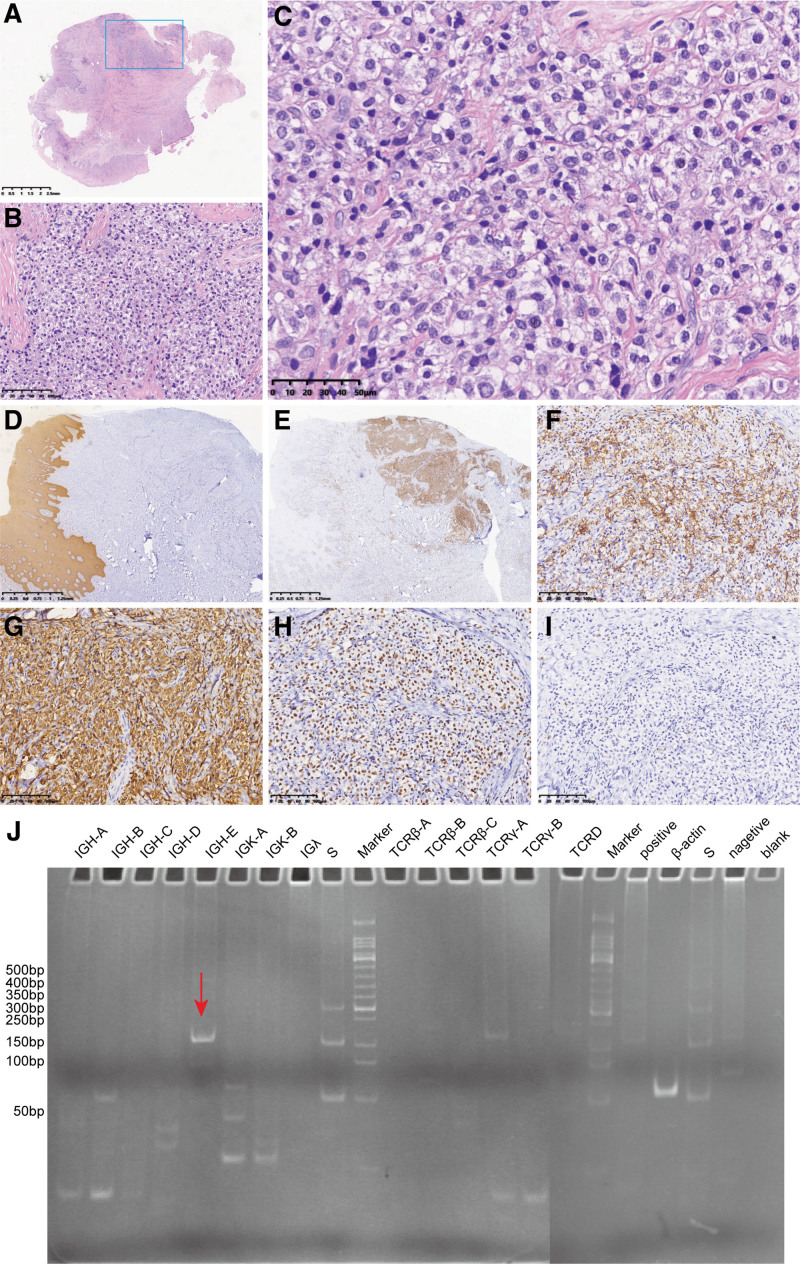
Microscopic histological and immunohistochemical features of a clear cell-like diffuse large B-cell lymphoma. (A, B) The tumor is located in the connective tissue beneath the mucosal layer. Low magnification shows tumor cells separated by fibrous tissue in nests and sheets. (C) Tumor cells are irregularly polygonal or ovoid, medium in size, with round or ovoid nuclei and small nucleoli visible under high magnification. (D) Immunohistochemical staining of a clear cell-like diffuse large B-cell lymphoma showing the tumor cell negative for cytokeratin AE1/AE3 (squamous epithelial cells are strongly immunopositive) and positive for (E) LCA (most tumor cells are strongly immunopositive), (F) CD20 (a substantial proportion of tumor cells are strongly immunopositive), (G) CD79a (most tumor cells are strongly immunopositive), (H) PAX5 (most tumor cells are strongly immunopositive). However, the tumor cells are negative for the T-cell marker (I) CD3. (J) The polyacrylamide gel electrophoresis map showing the presence of B-cell monoclonal rearrangement (DH7-JH) in the tumor (swim lane 5, red arrow).LCA = leukocyte common antigen.

A 44-year-old man was admitted to the First Affiliated Hospital of Kunming Medical University, complaining with 1-year’s history of an oral tumor, recently the tumor has increased and presented with enlargement of the right side of the face, slight difficulty in opening the mouth, with no other clinical symptoms. No unexplained fever, generalized swelling of the lymph nodes, loss of weight, etc. The patient denies any past medical history and family history of infectious or genetic diseases. The patient denied any previous medical history and family history of infectious or genetic disease. The Epstein-Barr virus (EBv), human Herpes virus 8 (HHV8) and human immunodeficiency virus (HIV) infection were not detected. On admission, the patient had normal temperature (36.4°C) and blood pressure (120/70 mm Hg). Oral examination revealed a hard and relatively firm mass of irregular size of approximately 1.0 cm. The mass was tender to palpation and skin surface without ulceration, and the systemic superficial lymph nodes were not enlarged. There were no other abnormalities were found during the general examination. Computed tomography (CT) showed an osteolytic lesion with soft tissue density in the right body of mandible with bone destruction in the cortical plates on both buccal and lingual sides (Fig. 1). The patient underwent a CT scan of the chest, which did not show an occupying lesion. The surgical biopsy tissue gross examination showed a piece of grayish mucosal tissue measuring 1.0 cm × 1.0 cm × 0.3 cm, with a grayish solid area of medium texture. Microscopically, the lesion was found to be located in the submucosal connective tissue with nested sheets of tumor cells. The tumor cells were epithelioid in appearance, with translucent cytoplasm and clear cell borders, of medium size, with round or ovoid nucleus and small visible nucleolus (Fig. 2A–C). We initially thought it might be clear cell type squamous cell carcinoma or neuroendocrine tumor, so we performed immunohistochemical tests related to epithelial origin, myoepithelioma and paraganglioma, including cytokeratin AE1/AE3, cytokeratin 5&6, CD56, P40, P63, chromogranin A (CgA), synaptophysin (Syn), epithelial to mesenchymal transition (EMA), SMA, calponin, but the test results were negative. We then considered the possibility that it could be other tumors with clear cell morphology, such as anaplastic malignant melanoma, tumors of vascular origin, and metastatic malignancies, etc. Expanded the immunohistochemical assay to include Vimentin, HMG-box 10 (SOX-10), S-100, MART-1/Melan-A, HMB45, Spalt-like transcription factor 4 (SALL4), OCT3/4, PAX5, ERG, calponin, SMA, actin (smooth muscle), Thyroid transcription factor-1 (TTF-1), thyroglobulin, parathyroid hormone (PTH), paired-box 8 (PAX-8), special AT-rich sequence-binding protein-2 (SATB-2), FLI-1. However, they are all negative except for vimentin. Finally, we tested for the leukocyte common antigen (LCA). Unexpectedly, tumor cells were diffusely positive for LCA. Immunohistochemical indicators and gene rearrangements were then used to determine the lymphohematopoietic tumor type. The IHC results showed that CD10, CD20, CD38, B-cell lymphoma (Bcl)-2, multiple myeloma oncogene (MUC)-1, CD79a were strongly expressed (Fig. 2D–I), while the tissue was negative for CD45RO, CD3, B-cell lymphoma 6 (Bcl-6), CD138, CD5, CD19, CD15, CD21, CD30, CD68, kapa, lanmda. The Ki-67 proliferation index was 40%. Detection of Ig and TCR gene arrangements using the BIOMED-2 protocols^[3]^ and the polyacrylamide gel electrophoresis map showing the presence of B-cell monoclonal rearrangement (DH7-JH) in the tumor (Fig. 2J). The final pathological diagnosis was germinal center B cell-like (GCB) diffuse large B lymphoma of clear cell type.

The patient is currently undergoing chemotherapy and has been followed up for 1 year. The patient’s condition has improved. This was an outpatient who had a pathology biopsy at my hospital and, after receiving a diagnostic pathology report, traveled to another hospital for treatment. We were told by telephone that he was undergoing chemotherapy. However, the patient did not know the details of the chemotherapy regimen and did not undergo systematic tests such as bone marrow biopsy and systemic PET-CT.

The patient in this case report provided verbal informed consent for publication of this report. The reporting of this case conforms to CARE guidelines.

## 3. Discussion

Diffuse large B-cell lymphoma is a relatively common malignancy in the daily diagnostic work of pathologists. But, the histological pattern of this case presents as a clear cell epithelial tumor, and the patient had no B symptoms. Based on tumor growth site, histological features and clinical presentation, the diagnosis we consider may be clear cell type squamous carcinoma, myoepithelioma, paraganglioma, anaplastic melanoma, bone tumors or even metastatic tumors including metastatic pretended paracellular adenocarcinoma, metastatic medullary carcinoma of the thyroid and metastatic germ cell tumors. But the immunohistochemistry associated with these diseases is negative except for vimentin. Finally, we performed immunohistochemical tests for tumors of the lymphohaematopoietic system, which showed negative T-cell immunophenotypes CD45RO, CD3 and CD5, and positive B-cell markers CD20 and CD79a and IGH clonal rearrangements. This case was finally considered to be a B-cell lymphoma. On Review of his CT imaging data, the patient had bone destruction and no history of peripheral blood abnormalities such as Bence-Jones protein. Immunohistochemistry in this case was negative for CD138, kappa, lambda and MUC-1, although CD38 and MUM-1 were expressed, still ruling out a plasmacytoma. The absence of epidermal invasion and diffuse CD10 expression in this case ruled out lymphoma in the extra-nodal marginal zone of mucosa-associated lymphoid tissue. According to the literature, primary mediastinal large B-cell lymphoma is so histologically heterogeneous, often with coarse collagen fibers, and the tumor usually consists of large cells with abundant cytoplasm, well-defined borders, translucent cytoplasm and round to ovoid nuclei.^[4]^ To determine whether this was a primary mediastinal large B-cell lymphoma, the patient underwent a CT scan of the chest, which did not show an occupying lesion and therefore excluded this diagnosis. Primary bone diffuse large B-cell lymphoma (PB-DLBCL), NOS may rarely contain clear cytoplasm cells and signet ring cells, thus mimicking metastatic adenocarcinoma.^[5,6]^ It has been reported that lymphoma cells may obtain a spindle morphology as they infiltrate into osseous and soft tissues.^[7]^ In this case, the CT showed an osteolytic lesion with soft tissue density in the right body of mandible with bone destruction in the cortical plates, but the biopsy was superficial and the histological morphology did not show a spindle cell pattern. It is difficult to distinguish between primary DLBCL of the bone and lymphoma cells that have infiltrated the bone. The final diagnosis of diffuse large B-cell lymphoma was made in conjunction with the immunophenotype. According to Hans’ classification criteria,^[8]^ the case expressed CD10 and belonged to the GCB subtype of DLBCL.

Diffuse large B cell lymphoma (DLBCL) is a prevalent subtype of non-Hodgkin’s lymphoma (NHL) worldwide. According to the World Health Organization’s Classification of Neoplastic Diseases of Hematopoietic and Lymphoid Tissues, DLBCL accounts for 30% to 40% of adult NHLs.^[9,10]^ The WHO 5th classifies DLBCL into 17 subtypes.^[4,11]^ However, DLBCL can present with variable changes such as mesenchymal sclerosis, mucinous degeneration, and cell spindle degeneration in routine diagnosis. These types of morphology have no impact on the patient’s prognosis and are therefore classified as nonspecific types. In this case, the tumor cells are of the hyaline epithelial type, which may be a histological variant. Diffuse large B-cell lymphoma with clear cell features has only been reported in 3 cases in the literature (Table 1), with the 3 cases occurring in the lymph nodes,^[13]^ retroperitoneum^[14]^ and bladder_ENREF_14.^[12]^ In two of the cases, the tumor cells were separated by fibrous septa into nested sheets without necrosis, and in the other case, lamellar necrosis was seen but without fibrous septa. In 2 cases the tumor cells originated from a non-germinal center with high proliferative activity and in the other case from a germinal center with a low proliferative index, as detailed in Table 1. Fibrous interstitial hyperplasia may be a phenomenon that limits their growth, but DLBCL is subject to conversion from lower to higher levels.

**Table 1:** Information on clear cell type diffuse large B-cell lymphoma.

Reference	Age (yr)	Primary site	Presence of B symptoms	Histologic appearance of malignancy			Immunophenotype							Type
				Clear cytoplasm	Interfibrillar substance	Necrosis	CD20	CD79a	Bcl-2	BCL-6	MUM-1	CD10	Ki-67	
Khurana et al^[12]^	75	Urinary bladder	No	+	+	−	+	+	/	/	/	+	+	GCB
Manxhuka-Kerliu et al^[13]^	39	Lymph node of neck	Yes	+	+	−	+	+	+	−	30%+	/	80%+	Non-GCB
Xue et al^[14]^	43	Lower abdomen	No	+	−	+	+	+	−	−	++	−	70%+	Non-GCB
The case	44	Oral	No	+	+	−	+	+	+	−	90%+	+	30%	GCB

Diffuse large B-cell lymphoma of the clear cell type must be differentiated from a number of diseases. Clear type epithelial cell carcinoma, where the tumor consists of a single clear polygonal cell arranged in sheets, nests or cords, with round nuclei, infrequent nuclear divisions, a distinct glassy interstitium usually visible around the nest, and tumor cells expressing cytokeratin. Neuroendocrine tumors in which the tumor cells express neuroendocrine markers. Tumor of myoepithelial origin with tumor cells expressing the myoepithelial markers S-100, cytokeratin 5/6, P63. Tumors Vascular-derived where the tumor cells express vascular-derived markers such as CD34, ERG. Malignant melanoma is also a histologically heterogeneous tumor and can be identified by the melanoma markers Melan-A, HMB-45, SOX-10. Metastatic parathyroid tumors expressing PTH. Plasmacytomas of the clear cell type that express the post-germinal centre and plasma cell markers MUM-1, CD138, CD38, kappa, lambda and MUC-1. Lymphoma in the extra-nodal marginal zone of mucosa-associated lymphoid tissue, visible as epidermolysis bullosa and expressing B-cell markers but not CD10, CD5. This is a rare histomorphological form of lymphoma, which has the morphological characteristics of a clear cell epithelial tumor and opens up diagnostic ideas for the diagnosis of lymphoma.

The case is currently out of follow-up and no further treatment or prognosis can be obtained.

## 4. Conclusion

Diffuse large B-cell lymphoma is the most common non-Hodgkin’s B-cell lymphoma with a wide range of sites and marked heterogeneity at the genetic, molecular, immunophenotypic, histological and therapeutic levels. Diffuse large B-cell lymphoma with a clear epithelioid cell morphology is highly misdiagnosed, although it has little impact on patient prognosis. It is our hope that this case report is an inspiration for the diagnosis of lymphoma.

## Acknowledgments

This work was supported by National Natural Science Foundation of China (82060423, 82360523), Xingdian Talent Plan “Famous Doctor Special Project” (RLMY20220018), Science and Technology Innovation Team for Precision Pathological Diagnosis of Lung Malignant Tumours at Kunming Medical University (CXTD202210) and Characteristic Teams of First-class Disciplines at Kunming Medical University (2024XKTDTS11).

## Author contributions

**Conceptualization:** Huan Luo.

**Data curation:** Yilong Huang.

**Investigation:** Zhiyuan Wang.

**Validation:** Jian Shen.

**Writing – original draft:** Guiyun Li.

**Writing – review & editing:** Qiang Feng, Li Bian.
